# Muscle contraction and relaxation-response time in response to on or off status of visual stimulus

**DOI:** 10.1186/1880-6805-33-23

**Published:** 2014-08-01

**Authors:** Kengo Yotani, Hiroki Nakamoto, Sachi Ikudome, Atsumu Yuki

**Affiliations:** 1 National Institute of Fitness and Sports in Kanoya, 1 Shiromizu, Kanoya, Kagoshima 891-2393, Japan; 2 Kochi University, 2-5-1 Akebono, Kochi, Kochi 780-8520, Japan

**Keywords:** Simple-reaction task, Visual response, Electromyogram

## Abstract

**Background:**

It is unclear whether response time is affected by a stimulus cue, such as a light turned on or off, or if there are differences in response to these cues during a muscle contraction task compared with a muscle relaxation task. The objective of this study was to assess the response time of a relaxation task, including the contraction portion of the task, to a stimulus of a light turned on or off. In addition, we investigated the effect of the pre-contraction level on the relaxation task.

**Results:**

Contraction response time was significantly shorter during the light-on status than during the light-off status (*P* <0.01), and relaxation response time in each maximum voluntary contraction was significantly longer during the light-on status than during the light-off status (*P* <0.01). The relaxation response time became longer in order of 25% to 75% maximum voluntary contraction regardless of light-on or -off status, and was significantly longer than the contraction response time (*P* <0.05-0.01).

**Conclusions:**

This study found that as the contraction level increased, the relaxation response time became longer than the contraction response time regardless of light status. However, contraction response time or relaxation response time findings were opposite to this during the light-on status and light-off status: contraction response time became shorter in the light-on status than in the light-off status and relaxation response time became longer in the light-on status than in the light-off status. These results suggest that the length of each response time is affected by motor control in the higher order brain and involves specific processing in the visual system.

## Background

Motor reaction tasks are a reliable assessment of sensorimotor or cognitive function and are used extensively to evaluate neural processing mechanisms. Response time (RT) during these tasks reflects the time needed for processing a sensory stimulus. Measurement of RT using electromyogram (EMG) can assess processing time of the nervous system and measure elapsed time from stimulus cue to EMG onset (for example, contraction) [1,2] or termination (for example, relaxation) [3,4].

Previous studies have reported that the RT to the stimulus cue of a light turned on lengthens the time of the muscle relaxation task more than the time of the contraction task [5]. On the other hand, Goldstone et al. [6] showed that the RT of the contraction task shortens at the stimulus cue of a light turned on more than a light turned off. Humans change the excitability level in the higher order brain by observing behaviors and facilitating response movements for compatible tasks rather than for incompatible tasks [7-10]. If there is an association between a stimulus cue (light on or off) and each task (contraction or relaxation), the RT in the relaxation task might be shorter during the stimulus cue of a light turned off than at a light turned on. However, it is not known whether RT is affected by such a stimulus cue.

The objective of the present study was to assess the RT of a relaxation task, including the contraction portion of the task, to a light turned on or off. In addition, we also investigated the effect of the pre-contraction level on the relaxation task.

## Methods

### Participants

Eight healthy male college students aged between 21 and 29 years participated in this study. All participants provided informed consent, and the study protocol was approved by the ethics committee of the National Institute of Fitness and Sports in Kanoya.

### Experimental procedures

**Figure 1:**
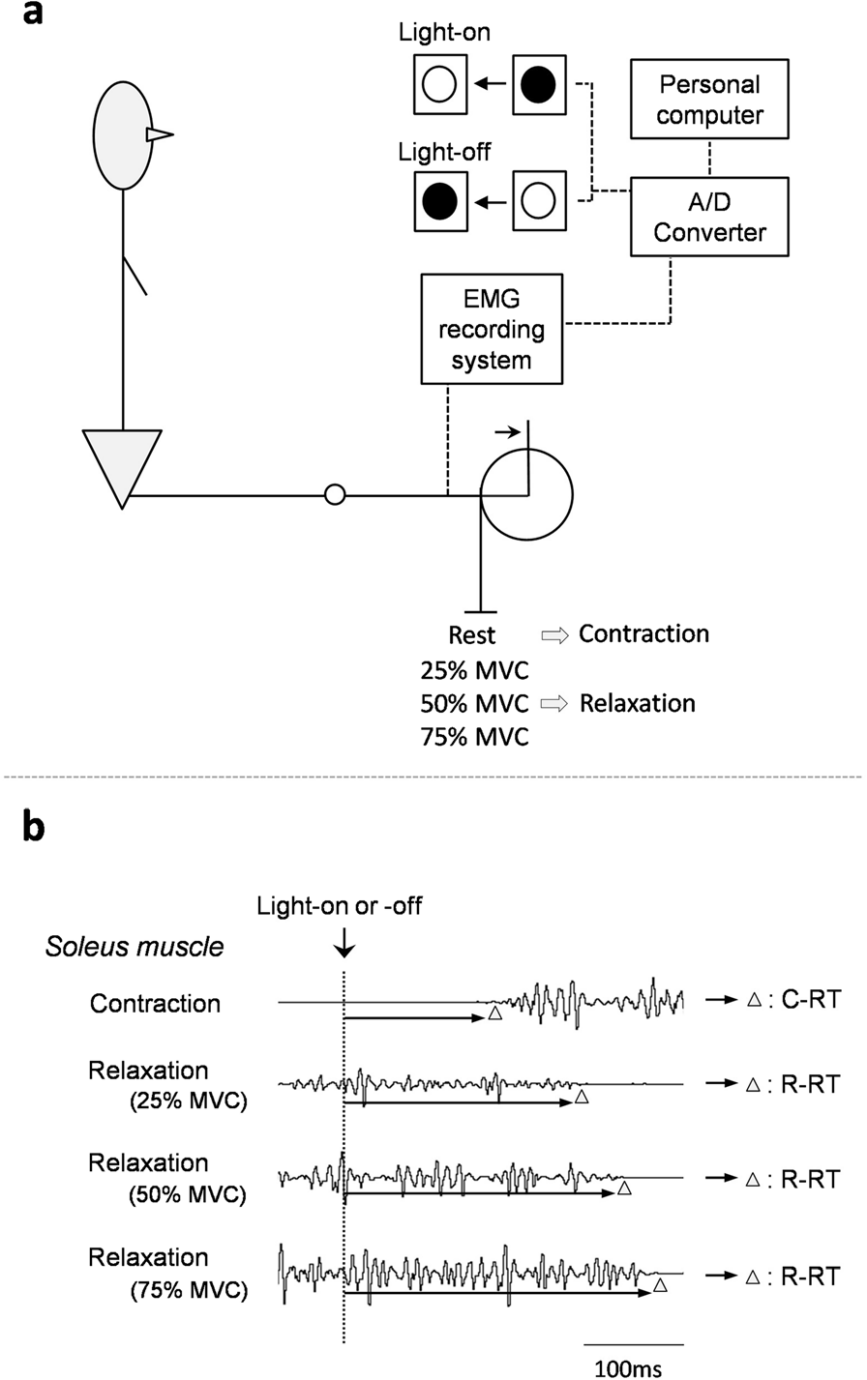
**Study schematic.** Schematic of experimental set-up **(a)**, and an example of EMG recording in soleus muscle during contraction or relaxation tasks during light-on status or light-off status (dotted line) **(b)**. Triangles indicate onset or termination of EMG signal. C-RT, contraction response time; EMG, electromyogram; MVC, maximum voluntary contraction; R-RT, relaxation response time.

Participants were seated comfortably in a custom-designed chair with the right leg fully extended. A visual signal was positioned by a red light-emitting diode (LED) at eye height and 1 m distance. The right foot was placed on a footplate attached to a resistance arm, which was counterbalanced at ankle joint angles of 90° of plantar flexion (that is, where the foot is considered to be perpendicular to the leg); the ankle joint was immobilized. Following the onset (on) or termination (off) of light, participants were asked to perform either an isometric muscle contraction, against the footplate, as quickly as possible (contraction task) or a quick release from a voluntary muscle contraction (relaxation task) (Figure 1). When performing the release, participants were specifically instructed not to activate the antagonist muscle. The light signal was turned on and off at pseudorandom intervals (2 to 4 s) after a warning signal. The relaxation tasks used three contraction levels, that is, 25%, 50%, or 75% of the maximum voluntary contraction (MVC), and light signal was administered only if a stable tonic was achieved at each level. During the relaxation task, the inter-trial interval was always greater than 3 min to avoid the effect of fatigue. Participants were allowed to practice until they became familiar with the experimental procedure and then performed 12 trials of each task during eight series (1 ‘contraction’ and 3 ‘relaxation’ tasks in the light-on or light-off condition, randomly mixed).

### EMG recording

EMG activity from over the lateral surface of the right soleus muscle was recorded using EMG electrodes. The soleus muscle was chosen because it is a single-joint muscle [11]. A DL 141 single-differential, parallel-bar configuration (4 Assist, Inc., Japan) was put on the skin surface over the muscle. The EMG signal was filtered during acquisition with a bandwidth of 5 to 500 Hz and a gain of × 1,000. The signals were digitized at 2 kHz (16 bit, PowerLab, AD Instruments, Japan). Onset and termination of EMG activity were recorded and stored for off-line analysis (Chart 6, AD Instruments) on a personal computer.

### Statistical analysis

The contraction response time (C-RT) was measured from the light-on or light-off status to the onset of EMG activity. The relaxation response time (R-RT) was measured from the light-on or light-off status to the termination of EMG activity. In the C-RT, the onset of EMG activity was identified from EMG signals using the methods described by Yotani et al. [12]. The EMG signals were processed using full-wave rectification (time constant: 0.05 s), and baseline activity was determined from the 50 ms period prior to visual cue onset. The threshold was set at above 3 standard deviation of the mean of baseline activity. Muscle activity onset was defined as the first point at which the EMG exceeded this threshold for at least 12.5 ms (25 consecutive samples). If the muscle was determined to be active, we returned to the threshold (+3 standard deviation) again and adopted a point of less than 1.5 standard deviation to indicate the onset of EMG activity. Furthermore, in the R-RT, termination of EMG activity was defined (the full-wave rectified traces) as the time when EMG activity was consistently below a value corresponding to the mean level of EMG signal in the pre-analysis period of the contraction task [3,4]. For the 12 trials of each task, the maximal and minimal C-RT and R-RT data were omitted to prevent anticipatory responses or possible inattention. The mean RT of 10 trials was taken as the value for each participant. All data were expressed as mean ± standard error and were analyzed using two-way analysis of variance (ANOVA) followed by Tukey’s post hoc test for comparison of C-RT and R-RT. Results were considered significant at *P* <0.05.

## Results

**Figure 2:**
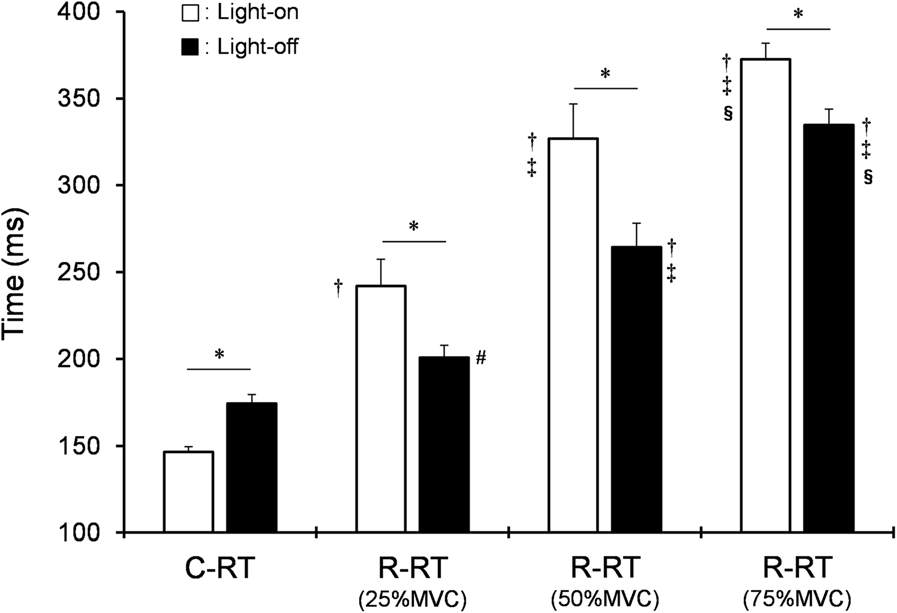
**Mean (±standard error) response time during C-RT and R-RT tasks.** * *P* <0.01, light-on status *vs.* light-off status; ^†^*P* <0.01, C-RT during light-on or -off status *vs.* R-RT of 25% to 75% MVC during light-on or -off status; ^‡^*P* <0.01, R-RT of 25% MVC during light-on or -off status *vs.* R-RT of 50% 75% MVC during light-on or -off status; ^§^*P* <0.01, R-RT of 50% MVC during light-on or -off status *vs.* R-RT of 75% MVC during light-on or -off status; ^#^*P* <0.05, C-RT *vs.* R-RT of 25% MVC during light-off status. C-RT, contraction response time; MVC, maximum voluntary contraction; R-RT, relaxation response time.

Figure 2 shows C-RT and R-RT in each MVC (25% to 75%). Two-way analysis revealed a significant main effect (light-on *vs.* light-off; *F* = 5.87, *P* <0.05, and C-RT *vs.* R-RT; *F* = 161.64, *P* <0.01). C-RT was significantly shorter during the light-on status than during the light-off status (*P* <0.01), and R-RT in each MVC was significantly longer during the light-on status than during the light-off status (*P* <0.01). In addition, the R-RT became longer in order of 25% to 75% MVC regardless of light-on or -off status, and was significantly longer than the C-RT (*P* <0.05-0.01).

## Discussion

There were two main findings in the present study. First, the stimulus cue of light (on or off) had a different effect on C-RT and R-RT. Second, with increases in contraction level, R-RT became longer than C-RT.

The optic nerve has on-fibers and off-fibers that respond to the on and off status of light, respectively [13]. A previous study using electroretinograms reported that the latency of response was shorter with light-on than with light-off [14]. Our results regarding the C-RT might reflect the effect of these latencies [6], whereas our R-RT findings are unlikely to be explained by this latency. The posterior parietal cortex is concerned with visuo-motor integration [15] and interacts with the cerebellum, which is critically associated with and mimics sensory-motor states [16]. Thus it seems likely that the visuo-motor process is associated with input from each optic nerve. Therefore, the length of C-RT (or R-RT) during the light on status (or off status) might involve not only afferent activity but also higher-order brain processing.

Our second key finding was that R-RT became longer than C-RT as contraction levels increased. The distal muscles have a larger cortical representation, and a previous study underlined the functional relevance of the powerful monosynaptic projections of these muscles [17]. Studies of movement-related cortical potentials and functional neuroimaging have shown that voluntary muscle relaxation is preceded and accompanied by activation of primary and supplementary motor areas [18-22]. In addition, as the neuronal clusters are connected to the primary motor cortex, it appears that these connections exert an inhibitory influence, at least in part [23,24]. The inhibitory influence is generally believed to test excitability of the GABA_A_-ergic system in the motor cortex, as well as short-interval intracortical inhibition (SICI). Indeed, the SICI decreases just before the onset of a voluntary contraction [25]. Hence removing ongoing inhibition can contribute to the increased excitability of the cortical motor pool, and the opposite process would occur with voluntary relaxation. Toma et al. [22] suggested that there is an increase in the activity of intracortical inhibitory interneurons that do not generate potentials. Our results showed that R-RT became longer than C-RT as contraction levels increased. Considering the report that R-RT of 30% MVC became longer than C-RT in crural muscle [5], voluntary muscle relaxation with an increase in contraction level might produce a greater time-lag between the decrease of motor output and the increase of inhibitory activity.

## Conclusions

In conclusion, this study found that as the contraction level increased, the R-RT became longer than the C-RT regardless of light status. However, C-RT or R-RT findings were opposite to this during the light-on status and light-off status: C-RT became shorter in the light-on status than in the light-off status and R-RT became longer in the light-on status than in the light-off status. These results suggest that the length of each RT is affected by motor control in the higher order brain and involves specific processing in the visual system.

## Abbreviations

C-RT: Contraction response time; EMG: Electromyogram; MVC: Maximum voluntary contraction; R-RT: Relaxation response time; RT: Response time; SICI: Short interval intracortical inhibition.

## Competing interests

The authors declare that they have no competing interests.

## Authors’ contributions

KY designed and carried out the study, performed the statistical analysis, and wrote the manuscript. SI helped with the data measurement of subjects. AY and HN helped with drafting the study design, assisted with data measurement, and interpreted the data. All authors read and approved the final manuscript.
